# Efficacy and safety of platelet-rich plasma in the treatment of severe burns: A protocol for systematic review and meta analysis

**DOI:** 10.1097/MD.0000000000023001

**Published:** 2020-11-06

**Authors:** Zhao Chen, Yuyan Wu, Nurlan Turxun, Yingjie Shen, Xue Zhang

**Affiliations:** a People's Hospital of Xinjiang Uygur Autonomous Region, Urumqi830001, Xinjiang Uygur Autonomous Region; b Linqing People's Hospital, Linqing252600, Shandong province; c Beijing Chaoyang Hospital affiliated to Capital Medical University, Beijing, China.

**Keywords:** effectiveness, platelet-rich plasma, protocol, severe burn wound, systematic review

## Abstract

**Background:**

In recent years, platelet-rich plasma (PRP) has been used in burn wound repair, plastic surgery, bone and tendon ligament injury repair and other treatment at home and abroad. Clinical studies indicate that PRP has a good curative effect on repairing burn wounds. The residual wounds formed after large area severe burns are difficult to cure and have recurrent attacks. Because the action mechanism of PRP is unclear, its clinical efficacy of PRP in repairing severe burn wounds is controversial. And there is no relevant systematic evaluation of PRP in repairing severe burn wounds now.

**Objective:**

Meta analysis was used to evaluate the efficacy and safety of PRP in repairing severe burn wounds.

**Methods:**

Randomized controlled clinical trial of using PRP to repair severe burn wounds were retrieved by computer WanFang, China National Knowledge Infrastructure, China Science and Technology Journal Database, China Biology Medicine disc, Embase, PubMed, Web of Science and Cochrane Library from the establishment of the database to September 2020. Two researchers independently extract data and assess the quality of the included literature, and Meta analysis of the included literature is carried out by using RevMan5.3 software.

**Results:**

In this study, the efficacy and safety of PRP in repairing severe burn wounds are evaluated from the aspects of wound healing rate, wound healing time, scar index, visual simulation score, the number of layers of dressing, the number of times of changing gauze, frequency of dressing change, the positive rate of wound bacterial culture and the incidence of inflammatory reaction.

**Conclusions:**

PRP has a good curative effect on the repair of severe burn wounds. This study provides reliable evidence for the clinical use of PRP in the clinical repair of severe burn wounds.

**OSF Registration number:**

DOI 10.17605/OSF.IO/FG682

## 1 Introduction

Burn refers to the tissue damage caused by heat, chemistry, electric current and other reasons, mainly refers to the skin and mucous membrane, and in serious cases can damage muscles, bones and even organs. Infection and organ damage after burn are the main causes of death of burn patients.^[1,2]^ Burns are the fourth most common traumatic injury in the human body, with an estimated 11 million people around the world seeking treatment for burns every year.^[3,4]^ Compared with mild to moderate burns,severe burns are more likely to leave scars, which may not only cause some dysfunction, but also seriously affect the image of patients, cause psychological burden and reduce the quality of life of patients.^[5,6]^ Current studies have shown that growth factor has a good effect on tissue repair of burn wounds.^[7,8]^

Platelet-rich plasma (PRP) is a kind of plasma rich Is a high concentration of plasma of platelets extracted from autologous anticoagulant blood by centrifugation.^[9]^ Platelets in PRP can release a variety of growth factors, promote the regeneration of tissue cells and stroma through different ways, and accelerate tissue repair.^[10,11]^ Platelets have bactericidal and antibacterial effects and can regulate wound repair by inducing proliferation of precursor cells and other cells.^[12,13]^ As the mechanism of action of PRP is still unclear, preparation standards and methods have not been unified, and the clinical efficacy of PRP in the repair of burn wounds is controversial. However, there is no relevant systematic evaluation of PRP for repairing severe burn wounds. Therefore, this study objectively evaluates the efficacy and safety of PRP in repairing severe burn wounds, and provides a scientific reference for the clinical application of PRP in tissue repair of severe burn wounds.

## 2 Methods

### 2.1 Protocol register

This protocol of systematic review and meta-analysis has been drafted under the guidance of the preferred reporting items for systematic reviews and meta-analyses. Moreover, it has been registered on open science framework on September 29, 2020. (Registration number: DOI 10.17605/OSF.IO/FG682).

### 2.2 Ethics

As this study is an analysis of the literature, there is no need to recruit patients, and the privacy of patients will not be disclosed, so patients’ informed consent and ethical approval are not required.

### 2.3 Eligibility criteria

#### 2.3.1 Types of studies

We will collected all available randomized controlled trails on PRP treatment for severe burn wounds, regardless of blinding, publication status, region, but Language will be restricted to Chinese and English.

#### 2.3.2 Research object

Patients with definite diagnosis of severe burn,^[14]^ regardless of their Chinese nationality, sex, age and location of the disease.

#### 2.3.3 Interventional measures

The treatment group is treated with simple PRP therapy, while the control group was treated with other treatment methods.

#### 2.3.4 Outcome indicators

Primary outcome: the wound healing rate;

Secondary outcomes: ① wound healing time; ② visual analog scale; ③ scar index; ④ frequency of dressing change; ⑤ Number of layers of wet gauze for dressing;⑥ Replacement times of inner gauze⑦ Positive rate of wound bacterial culture;⑧ Incidence of inflammatory response; ⑨ incidence rate of adverse reactions.

### 2.4 Exclusion criteria

(1) Duplicate published literature, and choose the 1 with the most complete data;
(2) If the paper is published as abstract or conference paper, the full-text paper cannot be obtained, and the data cannot be obtained by contacting the corresponding author;
(3) Studies with obvious data errors;
(4) Literature on the use of PRP combined with other treatments in the treatment group.

### 2.5 Search strategy

The key words “Fu xue xiao ban xue jiang(PRP),” “Shao shang(burn)” were searched in Chinese database, and “ PRP,” “platelet rich plasma,” “PRP,” “burn” and “bums” were searched in English database, including WanFang, China National Knowledge Infrastructure, China Science and Technology Journal Database, China Biomedical Database, Embase, PubMed, Web of Science, Cochrane Library, and so on. Randomized controlled clinical trial of using PRP to repair severe burn wounds were retrieved from the establishment of the database to September 2020. Take PubMed as an example, the retrieval strategy is shown in Table 1.

**Table 1:** Retrieval strategy of PubMed.

Number	Search terms
1	Platelet-rich plasma[Title/Abstract]
2	Platelet rich plasma[Title/Abstract]
3	PRP[Title/Abstract]
4	1 OR 2 OR 3
5	Burn[MeSH]
6	Burn[Title/Abstract]
7	Bums[Title/Abstract]
8	5OR 6 OR 7
9	4 AND 8

### 2.6 Data screening and extraction

**Figure 1:**
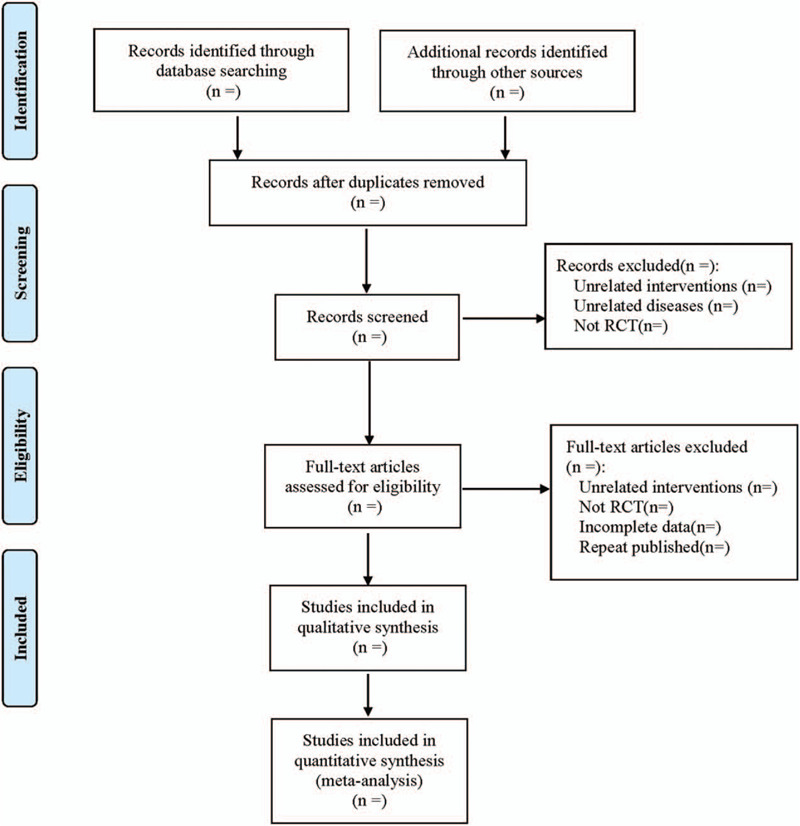
The process of literature screening.

Referring to the manual of systematic evaluation of Cochrane intervention measures and according to the the preferred reporting items for systematic reviews and meta-analyses flow chart, EndNote X7 software was used by 2 researchers to independently screen the retrieved literature based on the above inclusion and exclusion criteria. and negotiated with each other would be decided by the third researcher if there was a dispute over whether the study was included or not. Using the extraction table designed in advance to extract the research content, including① Basic data: title, author, publication date, source; ② research characteristics: number of cases, general demographic characteristics, intervention measures, follow-up, adverse events and so on; ③ Indexes of outcome: wound healing rate, wound healing time, visual analog score, score, scar index, frequency of dressing change, layers of wet gauze of dressing, changing times of inner gauze, positive rate of wound bacterial culture, incidence of inflammatory reaction and incidence of adverse reaction. The screening process is shown in Figure 1.

### 2.7 Literature quality evaluation

The methodological quality of the included study was evaluated according to the bias risk assessment tool in Cochrane5.1.0. From the 6 aspects of Random sequence generation, Allocation concealment, Blinding of participants and personnel, Blinding of outcome assessment, Incomplete outcome Data, Selective reporting, Other bias, the 2 researchers gave the judgment of “low risk, high risk, unclear” to cross-check assessment results, If differences are discussed and no agreement can be reached, it will be agreed with the third researcher and RevMan5.3 will be used for the risk assessment of bias.

### 2.8 Statistical analysis

RevMan5.3 software was used for Meta analysis. The counting data were compared using relative risk. If the measurement tools are consistent with the measurement units, the measurement data are expressed by the weighted mean difference, while the standard meandifference is used for interpretation. All effect quantities are represented as 95% confidential Interval. Heterogeneity test was performed by χ^2^ and *I*^*2*^ values, Heterogeneity test was performed by chi-squared and Chi-squared values, and the results (*P*≥0. 1, *I*^*2*^≤50%) showed good homogeneity among studies, and a fixed-effect model was adopted. If (*P<*0. 1, *I*^*2*^>50%) indicates heterogeneity among studies, analyzes the source of heterogeneity, excludes obvious clinical and methodological heterogeneity, and use the random effect model Subgroup analysis was used to analysis and process clinical heterogeneity, and only descriptive analysis was performed if there was significant clinical heterogeneity.

#### 2.8.1 Dealing with missing data

If there is data missing in the article, contact the author through the email to get the relevant data. If the author cannot be contacted, or the author has lost the relevant data, then do not carry out meta analysis and descriptive analysis will be conducted.

#### 2.8.2 Subgroup analysis

According to the dosage form of blood-rich platelet plasma, the subgroup was divided into 2 subgroups: the second degree and the third degree according to the depth of the burn, and the subgroup analysis was performed according to the course of treatment.

#### 2.8.3 Sensitivity analysis

In order to ensure the stability of the outcome index results, the sensitivity analysis of each outcome index was performed.

#### 2.8.4 Assessment of reporting biases

When the number of articles with outcome index was greater than or equal to 10, the funnel chart is used to evaluate the publication bias. In addition, Egger and Begg test were used for the evaluation of potential publication bias.

## 3 Discussion

PRP is a platelet concentrate obtained by whole blood centrifugation. Platelets release a variety of growth factors after activation, such as transforming growth factor β, fibroblast growth factor, platelet-derived growth factor, insulin-like growth factor, epidermal growth factor, vascular endothelial growth factor, keratinocyte growth factor, interleukin-8, and so on. These growth factors promote and regulate cell proliferation, adhesion, differentiation, mitosis, angiogenesis, collagen synthesis and secretion through multiple channels to achieve the purpose of accelerating tissue repair.^[15–18]^ In addition, studies have shown that PRP has a pain-relieving effect.^[19,20]^

Burn wound is the main cause of bacterial infection in patients with severe burn. Burn wound leads to the disappearance of skin barrier and denatured necrotic tissue provides good conditions for bacterial reproduction, which result in wound infection. Once the infection occurs, it often accompanies the whole course of the disease. until the wound healed completely.^[21,22]^ Related studies have shown that PRP can significantly repair severe burn wounds, while it is effective in the treatment of tissue infections such as bone infection.^[23,24]^ Therefore, on the 1 hand, the application of PRP in severe burn wounds can repair the wound and reduce the generation of bacteria, on the other hand, it can prevent the invasion and infection of bacteria, thus reducing the occurrence of wound infection.

PRP has a good effect on the repair of severe burn wounds, but because the mechanism of PRP is unclear, the standard method of preparation has not been unified, and its clinical effect in repairing severe burn wounds is controversial. Therefore, it is necessary to analyze the existing randomized controlled trail studies of PRP repairing severe burn wounds and objectively evaluate the clinical efficacy and safety of PRP in the treatment of severe burn wounds. However, due to the influence of the number of included studies and the quality of literature, this systematic evaluation still has its limitations. At the same time, subject to the language ability, we only searched the Chinese and English literature, ignoring the research of other languages. Therefore, more high-quality randomized controlled trials are needed to further confirm the efficacy and safety of PRP.

## Author contributions

**Data collection**: Nurlan Turxun and Yingjie Shen.

**Funding support**: Xue Zhang.

**Software operating**: Zhao Chen and Yuyan Wu.

**Supervision**: Xue Zhang.

**Literature retrieval**: Zhao Chen and Yuyan Wu.

**Writing – original draft**: Zhao Chen and Yuyan Wu.

**Writing – review & editing**: Zhao Chen and Xue Zhang.
